# An investigation of the umbilical artery N-terminal proBrain natriuretic peptide levels of fetuses due to fetal distress in term pregnancies

**DOI:** 10.1590/1806-9282.20240446

**Published:** 2024-08-16

**Authors:** Derya Erturk, Meryem Busra Birsen, Durmus Onder, Metin Kaba, Hamit Yasar Ellidag, Zeynep Ozturk Inal

**Affiliations:** 1 Antalya Training and Research Hospital, Department of Obstetrics and Gynecology – Antalya, Turkey.; 2 Antalya Training and Research Hospital, Department of Biochemistry – Antalya, Turkey.; 3 Konya City Hospital, Department of Obstetrics and Gynecology – Konya, Turkey.

**Keywords:** Fetal distress, Hypoxia, NT-proBNP, Pregnancy

## Abstract

**OBJECTIVE:**

This study aimed to investigate umbilical artery N-terminal proBrain natriuretic peptide (NT-proBNP) in fetuses delivered by cesarean section due to fetal distress in term pregnancies.

**METHODS:**

This prospective case-control study was conducted at the Antalya Training and Research Hospital Obstetric Department, Turkiye. A total of 140 pregnant women, 70 underwent elective cesarean sections between weeks 37 and 40 of gestation (Group 1, the control group) and 70 underwent cesarean sections due to fetal distress (Group 2, the study group), were included. The participants’ sociodemographic and obstetric data and fetal umbilical blood NT-proBNP levels were recorded in a database.

**RESULTS:**

Age, body mass index, gestational age, prenatal diagnostic tests, fetal anatomical scanning, and baby gender ratios were comparable between the groups (p>0.05), while statistically significant differences were observed in terms of gravidity (3.0 vs. 1.0, p≤0.001) and parity numbers (2 vs. 0, p≤0.001), baby height (50.36±0.88 vs. 49.80±0.86, p≤0.001) and weight (3422.43±409.16 vs. 3239.86±293.74, p=0.003), 1-min Apgar (9.0±0.1 vs. 8.5±1.3, p≤0.001) and 5-min Apgar (10.0±0.1 vs. 9.8±0.4, p=0.026) scores, umbilical artery pH (7.32±0.05 vs. 7.25±0.07, p≤0.001), umbilical artery base deficit (-2.48±1.23 vs. −4.36±1.09. p≤0.001), and NT-proBNP levels [8.77 (7.72–9.39) vs. 12.35 (9.69–12.92), p<0.001].

**CONCLUSION:**

This study showed that NT-proBNP can be used as an important marker in the diagnosis of fetal distress. Prospective studies with more participants are now needed to confirm the accuracy of our results.

## INTRODUCTION

Fetal distress is a syndrome involving respiratory and circulatory failure caused by intrauterine fetal hypoxia during birth and is closely associated with changes in fetal heart rate patterns. Fetal distress can cause hypoxic-ischemic encephalopathy and eventually lead to cerebral palsy and even perinatal death. Early detection and diagnosis of fetal distress can help prevent damage to the fetus's vital organs before birth^1–3^.

The natriuretic peptide family mainly consists of brain natriuretic peptide (BNP), C-type natriuretic peptide (CNP), and atrial natriuretic peptide (ANP) secreted from atrial myocytes. Although BNP was first isolated from pig brain tissue in 1988, later studies showed that it was largely synthesized and secreted from cardiac ventricular myocytes. It is mainly synthesized and secreted due to increased myocyte tension as a result of myocytes in the left ventricular wall being exposed to excessive pressure or increasing ventricular volume^4^.

BNP plays an especially important role in cardio-renal functions. It reduces sodium absorption in the proximal tubules and distal nephrons in the kidney and causes vasodilation by inhibiting renin, which causes vasoconstriction, thus resulting in diuresis and natriuresis. It also reduces the release of antidiuretic hormone and the synthesis and release of aldosterone, increases the glomerular filtration rate and renal blood flow, and reduces the release of endothelin by inhibiting the cardiac sympathetic system. BNP lowers cardiac preload and afterload to reduce stress on myocytes, leading to ventricular dysfunction in patients with acute coronary syndrome^5^. It has also been suggested that BNP is secreted to compensate for cardiac effects as a result of hypoxia. Pulmonary vasoconstriction, pulmonary hypertension, and overload on the right side of the heart occur in cases of fetal hypoxia. BNP and NT-proBNP are released from ventricular myocytes due to cardiac effects as a result^6^.

The aim of this study was to evaluate the umbilical artery NT-proBNP levels of fetuses delivered by cesarean section due to fetal distress in term pregnancies.

## METHODS

This prospective case-control study was conducted between 1 April and 1 December 2023, with the permission of the Health Sciences University Antalya Training and Research Hospital Ethics Committee, Türkiye (22-12-2022 and 23-3). A total of 70 pregnant controls who underwent elective cesarean sections (Group 1) and 70 women who underwent cesarean sections due to fetal distress between 37 and 41 weeks of gestation were included in the study. The two groups’ sociodemographic and obstetric characteristics and routine laboratory parameters were recorded in a database. Healthy pregnant women aged between 18 and 35 years at 37–41 weeks of gestation with no chronic diseases capable of adversely affecting fetal development (such as hypertension and systemic lupus erythematosus) were included. Fetal growth restriction, and congenital anomalies, and malformations detected in the fetus were excluded from the study.

### Fetal distress assessment

A fetal distress assessment was performed for the contraction stress test (CST). For the CST, a dilute oxytocin solution is infused until three contractions occur within 10 min. A positive (abnormal) test was accepted for late decelerations following ≥50 percent of contractions. The test was commented on as positive even if the contraction frequency was less than three in 10 min. The births of pregnant women with positive CST were performed by cesarean section because a positive (abnormal) CST indicates transient fetal hypoxemia during uterine contractions and may be an indication for delivery, depending on the clinical scenario.

### Serum N-terminal proBrain natriuretic peptide measurement

Fetal blood samples were obtained immediately after the fetal umbilical cord was clamped during the cesarean section; approximately 2 cc of sera were separated from the fetal cord blood. The samples were allowed to clot completely at room temperature and were centrifuged within 30 min at 3,000 rpm for 20 min. The samples were frozen at −80°C within 2 h and kept frozen until analysis.

A human NT-proBNP Elisa kit (Catalog No.: E1239Hu) was used to measure serum NT-proBNP levels. Sensitivity was studied using the enzyme-linked immunosorbent assay (ELISA) method with a standard curve range of 0.1–40 ng/mL and a sensitivity of 0.054 ng/mL. Plasma NT-proBNP levels were expressed as μg/mL.

### Statistical analysis

IBM Statistics Version 22 software was used for statistical evaluation. The normality of the distribution was evaluated using the Shapiro-Wilk test, with p>0.05 being regarded as a normal distribution. Normally distributed data were evaluated using the Student's t-test, and non-normally distributed data were evaluated using the Mann-Whitney U test. Cut-off values for NT-proBNP levels were determined using ROC analysis. The 95% confidence interval (CI) was calculated for the area under the curve (AUC). The sensitivity, the specificity, and the positive and negative predictive values of this variable were calculated in terms of its ability to predict fetal distress. Statistical significance was set at p<0.05.

## RESULTS

A total of 140 pregnant women, 70 underwent elective cesarean sections and 70 underwent cesarean sections due to fetal distress between 37 and 41 weeks of gestation, were prospectively enrolled consecutively in the study between 1 April and 1 December 2023.

The participants’ sociodemographic and obstetric characteristics and perinatal outcomes are presented in Table 1. Significant differences were observed in terms of gravidity [3.0 (2.0–4.0) vs. 1.0 (1.0–2.0), p<0.001], parity numbers [2.0 (1.0–2.0) vs. 0 (0–1.0), p<0.001], fetal length (50.36±0.88 vs. 49.80±0.86, p<0.001), birthweight (3422.43±409.16 vs. 3239.86±293.74, p=0.003), and 1-min Apgar (9.0±0.1 vs. 8.5±1.3, p<0.001) and 5-min Apgar (10.0±0.1 vs. 9.8±0.4, p=0.026) scores.

**Table 1:** The groups’ sociodemographic and obstetric characteristics and perinatal results.

			Control group (n=70)	Study group (n=70)	p
Age (years)			29.47±6.36	27.47±6.21	0.335
BMI (kg/m^2^)			30.73±4.09	31.02±3.12	0.645
Gravidity			3.0 (2.0–4.0)	1.0 (1.0–2.0)	<0.001*
Parity			2.0 (1.0–2.0)	0 (0–1.0)	<0.001*
Number of cesarean deliveries			1.0 (0–1.0)	–	
Gestational age (weeks)			39.1±1.1	39.2±1.3	0.512
Indication (n, %)	CPD		6 (8.6%)	0 (0%)	<0.001*
	Before cesarean delivery		21 (30.0%)	0 (0%)	
	PROM		0 (0%)	29 (41.4%)	
	Macrosomia		6 (8.6%)	0 (0%)	
	Recurrent cesarean delivery		34 (48.6%)	0 (0%)	
	Term pregnancy		0 (0%)	27 (38.6%)	
	Presentation abnormality		3 (4.3%)	0 (0%)	
	Surmaturity		0 (0%)	14 (20.0%)	
Double screening test (n, %)	Normal		36 (51.4%)	27 (38.6%)	0.126
Triple screening test (n, %)	Normal		29 (41.4%)	25 (35.7%)	0.487
Fetal anatomical screening (n, %)	Normal		41 (58.6%)	34 (48.6%)	0.236
OGTT (n, %)	Normal		31 (44.3%)	25 (35.71%)	0.301
Fetal height (cm)			50.36±0.88	49.80±0.86	<0.001*
Birth weight (g)			3422.43±409.16	3239.86±293.74	0.003*
Sex (n, %)		Female	37 (52.9%)	36 (51.4%)	0.866
		Male	33 (47.1%)	34 (48.6%)	
Apgar score		1-min	9.0±0.1	8.5±1.3	<0.001*
		5-min	10.0±0.1	9.8±0.4	0.026*
NICU admission (n, %)			0 (0%)	4 (5.7%)	0.120
NICU duration (days)			–	0 (0–0)	–

BMI: body mass index; CPD: cephalo-pelvic disproportion; PROM: premature rupture of membranes; OGTT: oral glucose tolerance test; NICU: neonatal intensive care unit.

* Statistically significant.

Maternal and fetal laboratory values are presented in Table 2. Serum Htc (34.17±3.33 in Group 1 vs. 35.67±4.20 in Group 2, p=0.021), WBC (9.84±2.13 vs. 11.31±2.77, p=0.002), and CRP [5.9 (3.5–9.3) vs. 9.2 (3.3–6.4), p=0.001] values differed significantly. Additionally, fetal umbilical artery pH (7.32±0.05 vs. 7.25±0.07, p<0.001), umbilical artery base deficiency (-2.48±1.23 vs. −4.36±1.09, p<0.001), umbilical artery pO_2_ (27.83±6.56 vs. 25.42±6.29, p=0.029), and umbilical artery NT-proBNP [8.77 (7.72–9.39) vs. 12.35 (9.69–12.92), p<0.001] levels differed significantly between the groups, while umbilical artery SatO_2_ and umbilical artery pCO_2_ were comparable between them.

**Table 2:** The groups’ laboratory values.

		Control (n=70)	Study (n=70)	p
MATERNAL	Hb (10³/mm³)	11.3±1.2	11.5±1.5	0.321
	Htc (10³/mm³)	34.17±3.33	35.67±4.20	0.021*
	WBC (10³/mm³)	9.84±2.13	11.31±2.77	0.002*
	PLT (10³/mm³)	228.64±59.60	251.23±75.31	0.051
	Glucose (mg/dL)	80.57±13.64	79.91±11.26	0.75
	Creatinine (mg/dL)	0.65±0.09	0.64±0.09	0.58
	ALT (U/L)	14.0 (10.0–17.0)	12.0 (9.0–16.0)	0.206
	AST (U/L)	15.0 (12.0–18.25)	16.5 (13.0–21.0)	0.142
	CRP (mg/L)	5.9 (3.5–9.3)	9.2 (3.3–6.4)	0.001*
	Leucocyte in urine	0 (0–2.75)	0 (0–4.0)	0.118
	Leucocyte esterase in urine	0 (0–0)	0 (0–0)	0.118
	Bacteria in urine	0 (0–0)	0 (0–0)	0.629
	Protein in urine	0 (0–0)	0 (0–0)	0.247
FETAL	Umbilical artery pH	7.32±0.05	7.25±0.07	<0.001*
	Umbilical artery base deficiency	-2.48±1.23	-4.36±1.09	<0.001*
	Umbilical artery SatO_2_	58.09±11.34	58.10	0.995
	Umbilical artery pCO_2_	41.87±6.81	42.95±8.93	0.423
	Umbilical artery pO_2_	27.83±6.56	25.42±6.29	0.029*
	Umbilical artery NT-proBNP (μg/mL)	8.77 (7.72–9.39)	12.35 (9.69–12.92)	<0.001*

Hb: hemoglobin; Hct: hematocrit; WBC: white blood cell; PLT: platelet; ALT: alanine amino-transferase; AST: aspartate amino-transferase; CRP: C-reactive protein; SatO_2_: oxygen saturation; pCO_2_: partial carbon dioxide; pO_2_: partial oxygen; NT-proBNP: N-terminal proBrain natriuretic peptide.

* Statistically significant.

Table 3 shows the sensitivity, specificity, positive predictive value (PPV), and negative predictive value (NPV) for umbilical artery NT-proBNP (AUC: 0.894; cut-off value: 8.54; CI: 0.800–0.927; sensitivity: 86%; specificity: 57%; PPV: 67%; NPV: 83%).

**Table 3:** Sensitivity, specificity, positive predictive value, and negative predictive values for umbilical artery N-terminal proBrain natriuretic peptide.

	AUC	Cut-off value	95%CI	Sensitivity	Specificity	PPD	NPD	p
Umbilical artery NT-proBNP	0.894	8.54	0.800–0.927	86%	57%	67%	83%	**<0.001** *

AUC: area under the curve; PPV: positive predictive value; NPV: negative predictive value; NT-proBNP: N-terminal proBrain natriuretic peptide.

* Statistically significant.

## DISCUSSION

This study showed that umbilical artery pH and pO_2_ levels decreased, base deficit increased, and fetal height, weight, and 1- and 5-min Apgar scores were lower in the fetal distress group. In addition, umbilical artery NT-proBNP levels were higher in the fetal distress group.

A previous study from Turkiye involving 130 pregnant women investigated the effect of the level of prenatal bonding between the mother and the fetus on prenatal outcomes. The authors reported that 49.2% of the participants had gravida 1 and 34.6% had gravida 2, while 62.3% had parity 0 and 30.8% had parity 1. The participants’ mean age was 30.37±4.75^7^. In the present study, the average age of the patients in the fetal distress group was 27.47±6.21, which is consistent with the literature. The median gravidity value was 1, and the median parity value was 0.

Jadhav et al. examined Doppler parameters in 32 pregnant women with fetal distress and 100 women who delivered by elective cesarean section. They found that the gravidity number of pregnant women who underwent an elective cesarean section was found to be higher (3.4±0.8 vs. 1.2±0.4) than that in the fetal distress group^8^. In another study involving multiparous and nulliparous women (nulliparous: 456 and multiparous: 152), the fetal distress rate was significantly higher in the nulliparas^9^. In the present study, gravidity and parity were both significantly lower in the fetal distress group.

Premature membrane rupture, which occurs spontaneously in 3% of women after the 37th week of pregnancy, can result in 85% neonatal morbidity and mortality. This condition was observed in 41.4% of the case group in the present study, the prevalence was 13.7% in Addisu et al.'s^10^ study.

Post-term pregnancy is associated with negative perinatal and maternal outcomes, and fetal morbidity and mortality increase in line with the gestation period^11^. A retrospective study from Iran of 8888 births between 2020 and 2022 reported a prevalence of 4.1%. For macrosomic babies, the odds ratio (OR) for premature birth was 2.24 (95%CI: 1.34–3.0), the OR for meconium amniotic fluid was 2.32 (95%CI: 1.59–3.14), and the OR for fetal distress was 2.38 (95%CI: 1.54–2.79)^12^. In the present study, premature pregnancies occurred in 20% of the fetal distress group, and we concluded that prolonging pregnancy exacerbates fetal distress, a finding consistent with the previous literature.

A study of the relationship between NICU requirements and fetal distress reported that fetal distress affecting birth weight and NICU requirements were higher in low birth weight babies^13^. In the present study, the height and weight of the babies in the fetal distress group were significantly lower than those of the control group babies, with 5.7% of the fetal distress group babies requiring NICU admission whereas none of the control group babies did.

Fetal acid-base balance affects the oxygenation of fetal organs, especially in the central nervous system and cardiovascular system, as well as Apgar scores^14^. The main buffers used to neutralize hydrogen ion production by the fetus are plasma bicarbonate and Hb. Although inorganic phosphates and erythrocyte bicarbonate also represent potential buffers, they play a less important role in fetal acid-base homeostasis^15^. In high-risk situations such as fetal distress, fetal acid-base balance is disrupted and meconium is observed in the amniotic fluid, resulting in changes in the fetal heart rate pattern^16^. A study of 216 pregnant women observed a decreased umbilical artery pH in the fetal distress group compared to the control group. In addition, a positive correlation was found between the level of acidosis in fetal umbilical artery pH and fetal hypoxia^17^. Bligard et al. reported that the risk of neonatal morbidity was twice as high in cases of fetal acidemia and that the risk of fetal mortality increased in line with the deepening of acidosis^18^. Consistent with the previous literature, significantly increased acidosis and higher base deficit were observed in the fetal distress group compared to the control group in our study, while no significant difference was observed in terms of partial carbon dioxide or saturation values.

BNP inhibits the cardiac sympathetic system in the circulatory system and reduces the release of endothelin. It also reduces the release of antidiuretic hormone and the synthesis and release of aldosterone in the urinary system, resulting in diuresis and natriuresis^19^. A study examining vasoactive and natriuretic mediators in umbilical cord blood reported BNP values between 4 and 17.4 pg/mL, with an average of 5 pg/mL^20^. Itoh et al. observed that umbilical artery BNP levels increased in newborns with fetal distress^21^. Vijlbrief et al. reported that BNP, whose median value was 69 pmol/L in umbilical blood samples from 164 newborns, was associated with fetal distress^22^. In the present study, the median umbilical artery NT-proBNP value of the fetuses in the fetal distress group was 12.35 μg/mL, higher than that of the control group.

Yadav et al. examined NT-proBNP levels in the intrauterine cord blood. The authors went on to suggest that NT-proBNP secretion increased as a result of myocardial insufficiency due to anemia. They also showed that NT-proBNP values were higher in patients with hydrops than in those without and were positively correlated with the degree of anemia^23^. In the present study, NT-proBNP levels increased significantly in the group that developed fetal distress as a result of fetal hypoxia.

Bayman et al. investigated the effect of gestational diabetes on BNP levels in the fetal umbilical artery. Although those authors determined no significant difference between the groups in terms of umbilical cord BNP levels, cord blood BNP values were higher in girls than in boys, and in the gestational diabetes (GDM) group using insulin compared to the non-insulin GDM group^24^. No significant difference was observed in BNP levels between male and female babies in the present study.

The fact that this study was conducted at a tertiary reference center represents an important limitation. However, a particular strength of the research is that the results from the study region can be adapted to all of Türkiye.

In conclusion, hypoxia and acidosis caused by stress increased the umbilical artery NT-proBNP values in newborns. Increased NT-proBNP levels resulting from changes caused by fetal distress may represent a harbinger of fetal morbidity and mortality. Prospective studies with larger numbers of participants are now needed to confirm the results of this study.
